# LFD implementation uncovers hidden rabies burden: Pre- and post-implementation analysis and comparison with non-implementation sites in the Philippines

**DOI:** 10.1016/j.onehlt.2025.101281

**Published:** 2025-11-19

**Authors:** Nobuo Saito, Patricia T. Lacanilao, Alyssa M. Garcia, Karren L. Inton, Jaira D. Mauhay, Voltaire G. Basinang, Lea G. Fernando, Benedict T. Bernardo, Roel G. Dela Cruz, Arvin H. Agapito, Arby B. Banaag, Gladys M. Bernardo, Jonarel M. Andres, Geraldine M. San Juan, Edwin P. Tecson, Annie M. Balingit, Joely T. Ongtangco, Maria G. Lagayana, Jeffrey L. Cruz, Shella G. Oridinario, Catalino S. Demetria, Daria L. Manalo, Beatriz P. Quiambao, Kazunori Kimitsuki, Akira Nishizono

**Affiliations:** a Research Center for Global and Local Infectious Diseases, Oita University, Yufu, Oita, Japan; b Department of Microbiology, Faculty of Medicine, Oita University, Yufu, Oita, Japan; c Institute of Tropical Medicine, Nagasaki University, Nagasaki, Nagasaki, Japan; d School of Tropical Medicine and Global Health, Nagasaki University, Nagasaki, Nagasaki, Japan; e Provincial Veterinary Office of Bulacan, Malolos, Bulacan, Philippines; f Municipal Agriculture Office of Pulilan, Pulilan, Bulacan, Philippines; g Municipal Agriculture Office of Guiguinto, Guiguinto, Bulacan, Philippines; h City Veterinary Office of San Jose del Monte, San Jose del Monte, Bulacan, Philippines; i Municipal Agriculture Office of Paombong, Paombong, Bulacan, Philippines; j Provincial Health Office of Bulacan, Malolos, Bulacan, Philippines; k Department of Agriculture, Regional Field Office III, Tarlac, Tarlac, Philippines; l Department of Agriculture, Bureau of Animal Industry, Quezon City, Metro Manila, Philippines; m Research Institute for Tropical Medicine, Muntinlupa, Metro Manila, Philippines

**Keywords:** LFD, One health, Implementation study, Outbreak, Time series analysis

## Abstract

**Background:**

Despite accumulating evidence supporting lateral flow devices (LFDs) in rabies diagnosis, their impacts of decentralizing diagnostic capacity on surveillance in endemic settings have not been systematically evaluated.

**Methods:**

We established low-resource decentralized rabies diagnostic laboratories in a target area of the Philippines, where rabies testing was previously unavailable, and evaluated both the diagnostic performance of LFD and their impacts on surveillance.

**Results:**

Using DFAT (direct fluorescent antibody test) as reference, LFDs performed at the decentralized laboratories showed a sensitivity of 97.0 % and a specificity of 98.2 %. After implementation, the monthly number of confirmed animal rabies cases in the target area increased from 2.09 to 8.65 cases, representing a 4.14-fold increase (*p* < 0.001), a change not observed in neighboring provinces without LFD implementation. Interrupted time series analysis further supported a significantly increasing trend in case detection after the implementation (coefficient = 0.565, p < 0.001), which was not observed in non-implementation provinces. Through 69 outbreak investigations triggered by LFD-positive cases, 22 unvaccinated human bite victims and 66 exposed animals were additionally identified. However, while case investigations were performed in 54.8 % of cases, integrated One Health investigations involving coordinated actions by local health and veterinary offices were conducted in only 5.6 %.

**Conclusion:**

LFD-based decentralization markedly improved case detection and enabled critical outbreak responses at the local level, demonstrating its value for rabies control in endemic settings though sustained One Health approach remains a challenge following the detection of rabid animals using LFD.

## 1 Background

Rabies is an almost invariably fatal zoonotic disease, with a nearly 100 % case fatality rate once clinical symptoms develop [1]. The disease remains endemic in many regions of Asia and Africa, where domestic dogs serve as the main reservoir and the primary source of human mortality [2]. In rabies-endemic areas, all individuals exposed to potentially rabid animals are recommended to receive Post-Exposure Prophylaxis (PEP), consisting of wound washing, rabies immunoglobulin, and a series of vaccinations [3]. The rising incidence of dog bites, particularly in densely populated countries in Southeast Asia, has dramatically increased the demand for PEP [[4], [5], [6]]. In the Philippines, over one million people visit healthcare facilities to receive PEP every year, posing a substantial burden on the health system [7]. Despite the enormous annual budget for PEP, its provision alone is insufficient to interrupt rabies transmission [8]. Rabies elimination is achievable through effective control measures targeting the canine reservoir, emphasizing the need to prioritize interventions and allocate resources accordingly [9,10].

For effective rabies control, accurate identification of rabid animals and systematic monitoring of local transmission dynamics are essential [11,12]. Confirmatory rabies diagnosis remains a significant challenge in many rabies-endemic regions of low- and middle-income countries (LMICs) [13]. Rabies testing requires brain samples from clinically suspected animals and uses standard methods such as the direct fluorescent antibody test (DFAT), direct rapid immunohistochemical test (DRIT), or reverse transcription polymerase chain reaction (RT-PCR) [14]. These methods depend on infrastructure that remains largely inaccessible in rabies-endemic regions of LMICs. Transporting highly infectious and decomposition-prone brain samples under cold chain and biosafety conditions remains a major challenge in remote areas. Even when samples reach diagnostic laboratories, it often takes several days to weeks for the results to reach the local level. These logistical constraints discourage sample submission, resulting in limited testing and widespread underreporting of rabies in many endemic areas [11,15]. The absence of reliable data prevents policymakers and the public from understanding the actual situation. To break this cycle, it is essential to improve the visibility of the rabies problem through accurate and rapid diagnosis.

Rabies lateral flow devices (LFDs) are simple tools that do not require specialized equipment and serve as a suitable alternative diagnostic tool to strengthen surveillance, particularly in areas where standard methods like DFAT are unavailable [[16], [17], [18], [19], [20]]. Further advantages of LFDs include easier transport, as positive kits can be used as samples for genomic analysis without requiring a cold chain [17,21]. LFDs also demonstrate high sensitivity with decomposed brain samples, unlike DFAT [22]. However, a primary limitation of LFDs is their inconsistent diagnostic sensitivity, which varies across commercially available kits, although problems with specificity are infrequent [16,17,19,[23], [24], [25]]. Despite their growing use, evidence on the impact of LFD introduction remains limited. In Zanzibar, the LFD introduction led to a fourfold increase in rabies case detection [20]. However, this analysis relied solely on a pre- and post-implementation comparison. Due to the fluctuating epidemic waves of rabies, such comparisons alone may not fully capture the impact of LFDs; thus, more robust evaluations incorporating parallel assessments with non-intervention areas are warranted to ensure accurate interpretation. This study evaluated the impact of LFD implementation on rabies surveillance by analyzing pre- and post-implementation time-series data, along with comparisons between areas with and without LFD implementation, to assess changes in case detection.

## 2 Methods

### 2.1 Ethics statement

The study was approved and supported by the 10.13039/501100003921Department of Health (DOH) and the 10.13039/501100003526Department of Agriculture–Bureau of Animal Industry (DA-BAI). All samples were collected postmortem from animals that died naturally or were euthanized due to suspected rabies, with euthanasia performed by local government veterinarians at their discretion. These activities were part of the official rabies surveillance and response program, as outlined in the national rabies guidelines of the Philippines [26]. We managed biohazard risks by following standard biosafety protocols, using personal protective equipment (PPE) and ensuring that all staff received pre-exposure rabies vaccination. The animal care and use aspects of the protocol were reviewed and approved by the RITM Institutional Animal Care and Use Committee (IACUC No. 2021–08).

### 2.2 Study sites

**Fig. 1:**
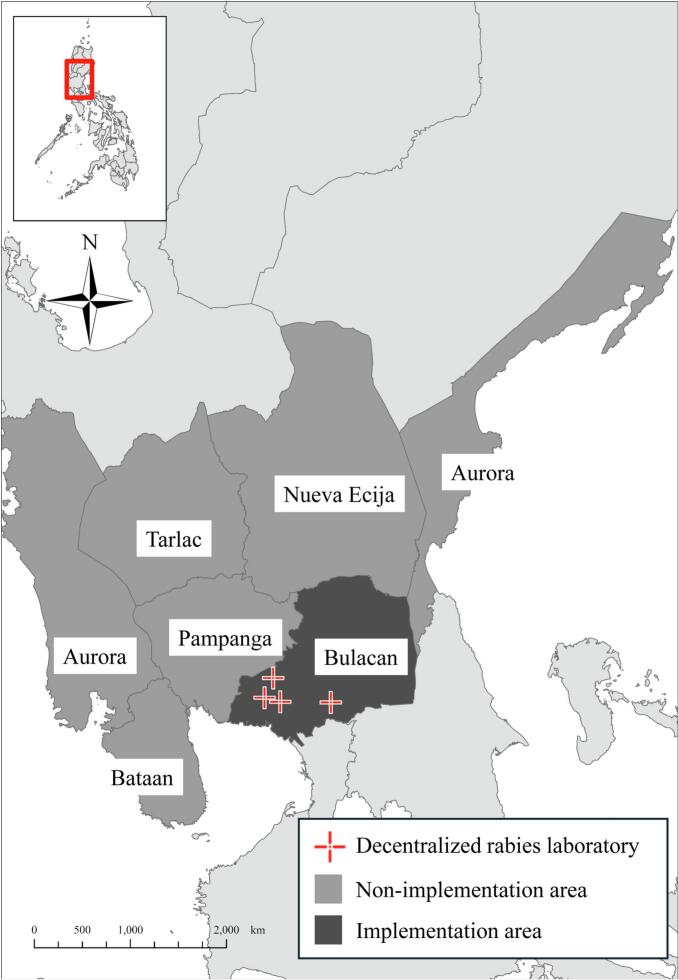
Geographic distribution of the LFD implementation area (Bulacan) and non-implementation areas in Region III, Philippines. This map shows Bulacan Province (dark gray), the designated LFD implementation area, and the surrounding provinces (light gray) in Region III, Philippines, which served as non-implementation areas for comparison. Red crosses indicate the locations of decentralized rabies laboratories. (For interpretation of the references to colour in this figure legend, the reader is referred to the web version of this article.)

We conducted the study in Bulacan Province, a peri-urban area north of Metro Manila with a population of 3.7 million and a high population density of approximately 1200 persons/km^2^ as of the 2020 census. Prior to project implementation, the province had no animal diagnostic laboratory, and suspected rabies samples had to be submitted to a regional facility in Pampanga Province, approximately two hours away by car (Fig. 1). Region III (Central Luzon) comprises seven provinces: Bulacan, Aurora, Bataan, Nueva Ecija, Pampanga, Tarlac, and Zambales, and consistently reports the highest number of human rabies cases in the Philippines, with 30 to 50 cases annually. Bulacan, the study site, is a rabies-endemic province, with approximately 10 human rabies cases reported each year (0.27 per 100,000 persons). However, due to the absence of an animal rabies diagnostic laboratory, detection and reporting of animal rabies cases in the province had been limited [13]. For this reason, Bulacan Province was selected as the implementation site for this study to improve animal rabies surveillance through decentralized diagnostic capacity. Within the province, decentralized rabies diagnostic laboratories were established in five local government units: Guiguinto Municipal Agriculture Office (MAO), Paombong MAO, Pulilan MAO, Bulacan Provincial Veterinary Office (PVO), and the City Veterinary Office (CVO) of San Jose del Monte (Fig. 1). These sites were selected based on their geographic distribution, accessibility, and the presence of designated personnel responsible for rabies response.

### 2.3 Implementation of decentralized rabies testing and use of RaDSS

Between April and September 2021, we implemented sequential training sessions in the five selected local government units to introduce LFD testing. In this study, we defined October to December 2021 as the introduction phase and January 2022 as the beginning of the implementation phase. Implementation in Paombong was delayed due to administrative issues, with diagnostic activities starting in November 2022. We also trained staff from veterinary services, public health offices, and Animal Bite Treatment Centers (ABTCs) to use RaDSS (Rabies Data Sharing System), a digital platform for surveillance and case reporting. RaDSS, developed by DA-BAI and Oita University, is operated by the information system unit of DA-BAI and integrated with the national veterinary database PHILAHIS (http://philahis.bai.gov.ph/). RaDSS enables rapid and coordinated responses following laboratory confirmation of animal rabies cases. We provided each site with LFD kits and essential supplies, including metal tables for sample handling, compact freezers for specimen storage, and PPE such as gowns, gloves, face shields, and surgical masks. The study also supplied animal cages and catch nets to support the safe handling and observation of suspected rabid animals. To promote timely reporting of suspected rabid animals, we distributed posters and leaflets to health offices, animal bite treatment centers (ABTC), and barangay health stations. These materials included contact information for the local veterinary offices and emphasized the importance of reporting animal deaths immediately, especially in cases involving bite victims. Following laboratory confirmation of animal rabies cases, rabies outbreak responses were recommended according to the national guidelines [26,27]. In line with these guidelines, our study promoted the same set of responses and included them in the training sessions provided to local personnel. These included (i) joint case investigation under a One Health approach, involving collaboration between the local veterinary office, the health office, and the barangay (the smallest administrative unit); (ii) contact tracing of potentially exposed animals and humans, followed by quarantine or provision of PEP as appropriate; (iii) public notification and risk communication; (iv) restriction of free-roaming animals; and (v) ring vaccination of dogs and cats within a 3-km radius from the confirmed case.

### 2.4 LFD testing at decentralized laboratories and evaluation of diagnostic accuracy

In this study, we performed all sampling and LFD testing at decentralized rabies laboratories, avoiding field-based procedures at the site of animal detection. Carcasses of suspected rabid animals and clinically suspected animals were transported to decentralized rabies laboratories, where diagnostic procedures were performed under controlled conditions. Following decapitation, we collected brain samples using the straw sampling method and tested them with the ADTEC LFD (Rabies Ag Test, ADTEC Co., Ltd., Oita, Japan; Lot Nos. 2103, 2104, and 2209), as described elsewhere [23,28]. We interpreted faint test lines as positive, as previously applied in LFD validation studies [19,23]. LFD results from decentralized laboratories were recorded monthly using a standardized reporting form and submitted to the research team. Decapitated heads were stored at −20 °C until batch shipment to the regional laboratory, where confirmatory testing by DFAT was performed. In cases with discrepant results between LFD and DFAT, we conducted additional testing using the LN34 RT-qPCR assay, as described elsewhere [23,29]. For the evaluation of diagnostic accuracy of LFD performed at decentralized laboratories, we excluded cases with invalid LFD results, missing head submission, or unavailable DFAT due to decomposition or inconclusive findings. Using DFAT as the reference standard, we determined the sensitivity and specificity of the LFD performed at the decentralized rabies laboratories. The study adhered to the guidelines outlined in the STARD (Standards for Reporting of Diagnostic Accuracy Studies) statement (see Supplementary Table S1).

### 2.5 Evaluation of the impact of decentralized rabies testing on case detection

To evaluate the impact of LFD implementation and to compare trends with other provinces in Region III, we used annual data on laboratory-confirmed animal rabies cases extracted from the official reports of the regional laboratory, with reported cases based on specimens submitted for routine DFAT under the passive surveillance system. We used only DFAT-confirmed positive cases for this analysis, as nearly all LFD-tested specimens in Bulacan were submitted to the regional laboratory for confirmatory DFAT. We obtained data from 2014 to 2023, defining 2022–2023 as the post-implementation phase and the preceding years as the pre-implementation phase. To evaluate the impact of the implementation on the monthly number of rabid animal cases, boxplots were generated to show the distribution of monthly rabies case counts by province during the pre- and post-LFD implementation periods. We compared monthly case counts between the pre- and post-implementation periods using the Mann–Whitney *U* test to evaluate differences in distributions and the two-sample *t*-test to assess differences in means. The fold change was calculated as a simple ratio of means (post/pre), using the pre-implementation period (2014–2021) and the post-implementation period (2022−2023).

We also performed interrupted time series (ITS) analysis using monthly DFAT-confirmed animal rabies cases from January 2014 to May 2023. The implementation period was divided into an introduction phase from October to December 2021 and a full implementation phase starting in January 2022. Considering a possible increase in rabies endemicity in 2022, as indicated by a nationwide rise in human rabies cases, we included a comparative component using three neighboring provinces (Pampanga, Tarlac and Zambales) that did not implement LFD testing but were assumed to have similar background rabies trends. We applied a generalized linear model with Newey–West standard errors to adjust for autocorrelation and heteroskedasticity. The model included terms for baseline trend, level change at implementation, and post-implementation trend, along with interaction terms to compare slopes between Bulacan and each control province.

To assess geographic changes in animal rabies detection, we created case maps showing the spatial distribution of DFAT or LFD confirmed animal rabies cases during the pre-implementation (2014–2021) and post-implementation (2022–2023) periods. Case locations were plotted using individual GPS coordinates recorded in RaDSS or obtained from the regional laboratory's reports. Spatial mapping was performed using ArcGIS Pro version 3.4.0 (ESRI, Redlands, CA, USA).

### 2.6 Investigation of rabies outbreak responses following LFD-positive cases

To examine the implementation status of rabies outbreak responses following LFD-positive diagnoses, we reviewed RaDSS entries submitted by local government veterinarians. Considering that some reports lacked sufficient detail, research staff conducted follow-up interviews by phone with relevant local stakeholders approximately four weeks after case confirmation to document response activities. Information on reporting delays, implementation of control measures (e.g., mass vaccination, community awareness activity, dog population management), and outcomes related to human and animal exposures was extracted and summarized.

## 3 Results

### 3.1 Diagnostic performance of LFD testing at decentralized laboratories

**Fig. 2:**
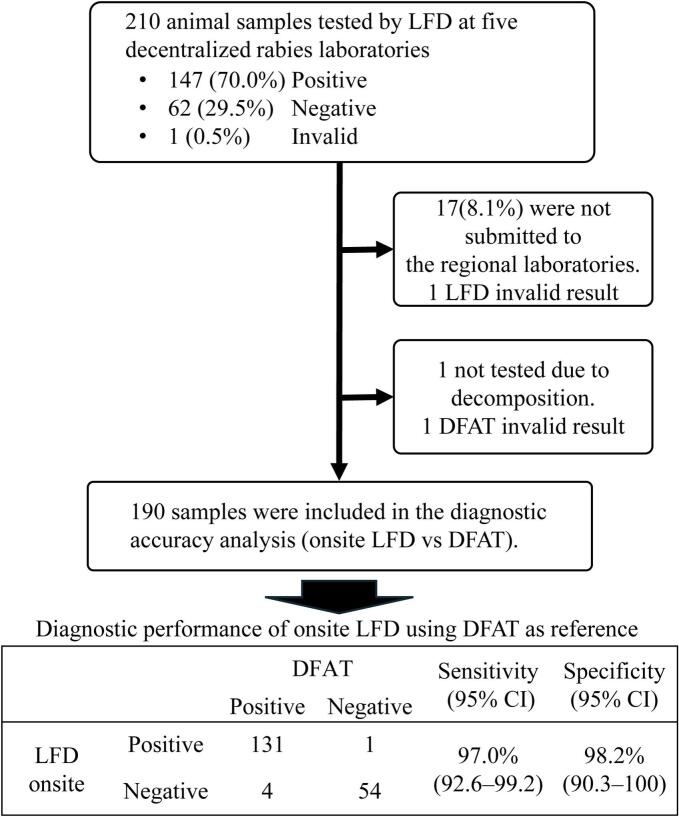
Flow diagram showing sample selection and diagnostic performance of onsite LFDs compared to DFAT. LFD, lateral flow device; DFAT, direct fluorescent antibody test; CI, confidence interval.

During the study period, five decentralized rabies laboratories tested 210 animal samples using LFDs. Most were dogs (188/210, 89.5 %), followed by cats (21/210, 10.0 %) and one goat (0.5 %). San Jose Del Monte CVO tested the most samples (94, 44.8 %), followed by Pulilan MAO (52, 24.8 %), Bulacan PVO (44, 21.0 %), Guiguinto MAO (15, 7.1 %), and Paombong MAO (5, 2.4 %). Among the 210 samples, 147 (70.0 %) were positive, 62 (29.5 %) were negative, and one (0.5 %) was invalid by onsite LFD. 193 (91.9 %) were subsequently submitted to the regional laboratory for confirmatory testing, while 17 (8.1 %) were not submitted (Fig. 2). At the regional laboratory, one sample was not tested due to decomposition, and one DFAT result was inconclusive. Excluding 17 unsubmitted samples, one invalid LFD result, one decomposed sample and one invalid result of DFAT, 190 samples were included in the diagnostic accuracy analysis. Among these, 135 were positive and 55 were negative by DFAT. Using DFAT as the reference, LFD showed a sensitivity of 97.0 % (95 % CI: 96.2–99.2) and a specificity of 98.2 % (95 % CI: 90.3–100) (Fig. 2). Among the samples with discrepant results between LFD and DFAT, five were further tested by LN34 RT-qPCR. Of these, two were determined to be false negatives by LFD, one appeared to be a false positive by LFD, and two were considered false positives by DFAT (see Supplementary Table S2).

### 3.2 Longitudinal trends in animal rabies detection pre- and post-LFD implementation

**Fig. 3:**
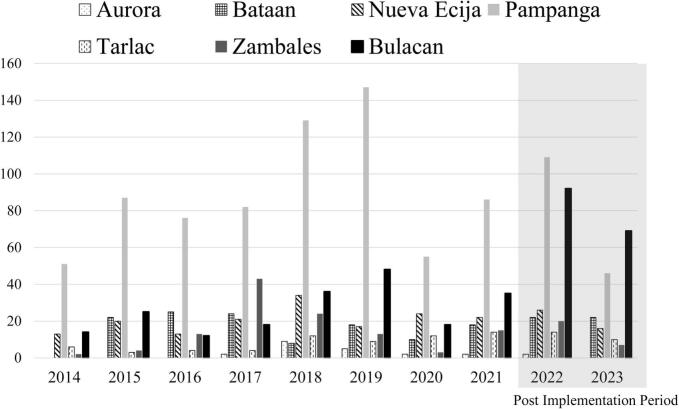
Annual animal rabies case detection in Bulacan (LFD implementation site) and surrounding provinces in the pre- and post-LFD implementation period. The shaded box indicates the period after the implementation of LFD-based testing at decentralized rabies laboratories, which began on January 1, 2022. Data represent confirmed cases reported from seven provinces in Central Luzon.

**Fig. 4:**
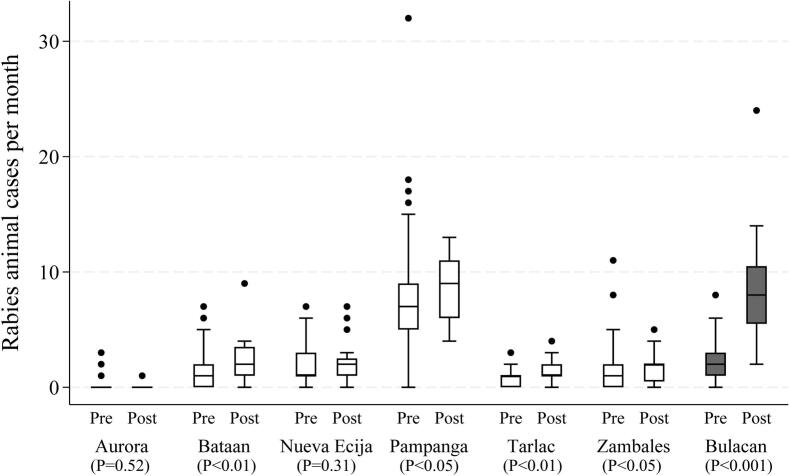
Average monthly rabies cases per province in the pre- and post-LFD implementation periods in Bulacan (LFD implementation site) and surrounding provinces. Boxplots display the distribution of monthly laboratory-confirmed animal rabies cases in each province during the pre-implementation period (January 2014–December 2021) and post-implementation period (January 2022–December 2023). The central line in each box represents the median, the box indicates the interquartile range (IQR), and the whiskers represent the minimum and maximum values within 1.5 × IQR. Dots represent outliers beyond this range. LFD was implemented only in Bulacan; all other provinces remained under standard surveillance protocols. Data are based on DFAT-confirmed cases reported by the regional laboratory. *P*-values shown below each province represent results of the Mann–Whitney *U* test comparing pre- and post-implementation periods.

**Fig. 5:**
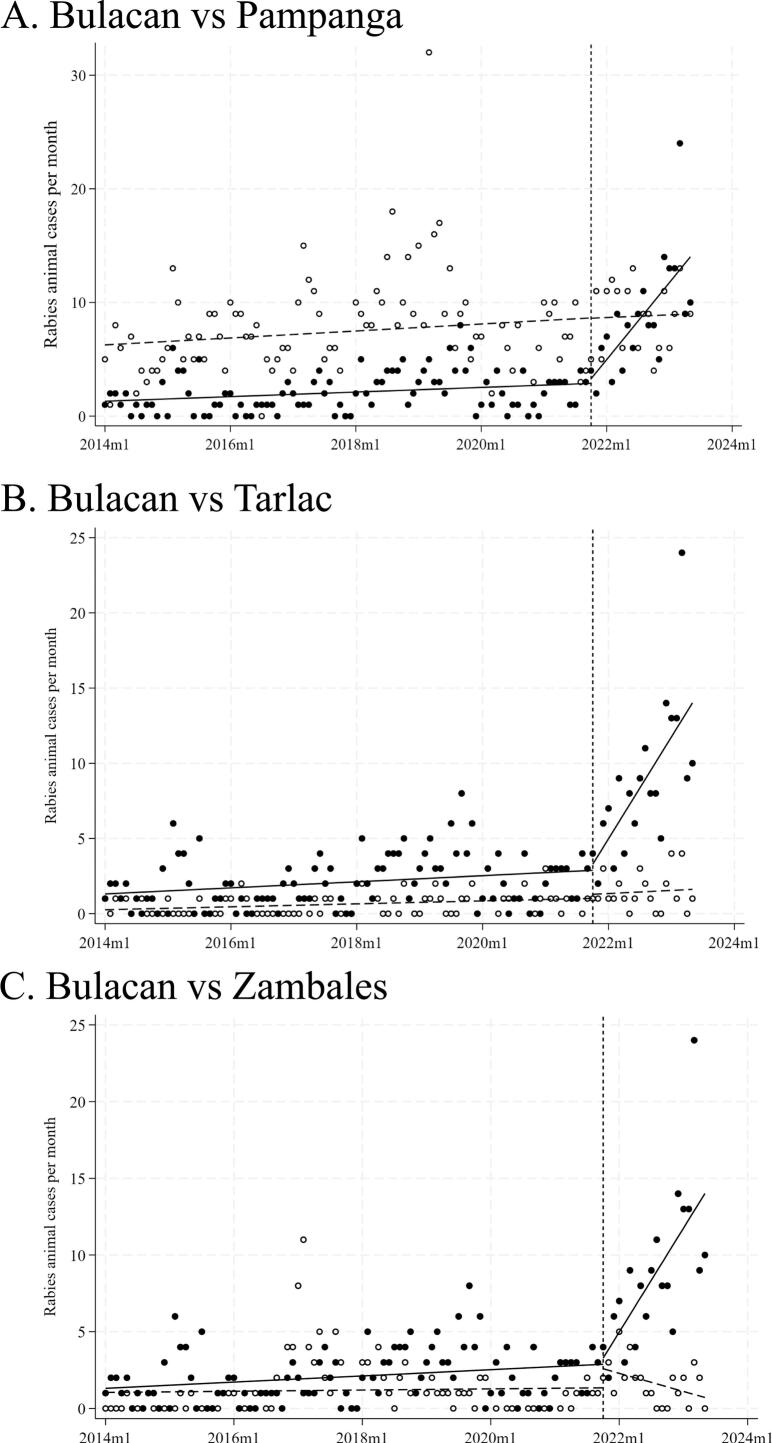
Monthly confirmed animal rabies cases in the LFD implementation area (Bulacan) and non-implementation areas (Pampanga, Tarlac, and Zambales): Interrupted time series analysis. This figure compares monthly DFAT-confirmed animal rabies cases between the LFD implementation area (Bulacan) and three non-implementation provinces (Pampanga, Tarlac, and Zambales). ITS was conducted with the implementation period defined as beginning in October 2021, including the introduction phase (October–December 2021). Solid circles (●) and solid trend lines represent observed cases and fitted trends in Bulacan; open circles (○) and dashed trend lines represent those in the comparison provinces. The vertical dashed line indicates the start of the implementation period.

Fig. 3 shows the annual number of laboratory-confirmed animal rabies cases in Bulacan, where LFD testing was implemented in January 2022, and in the surrounding non-implementation provinces. Confirmed rabies cases increased notably in Bulacan after implementation, particularly in 2022 and 2023, whereas no such increase was observed in neighboring provinces. The monthly median number of confirmed animal rabies cases pre and post LFD implementation is shown in Fig. 4. Based on the Mann–Whitney *U* test, a statistically significant difference (*p* < 0.05) in monthly case counts between the two periods was observed in Bataan, Bulacan, Pampanga, Tarlac and Zambales with Bulacan showing the most pronounced increase. In Bulacan province, the mean monthly case detection increased significantly from 2.09 to 8.65 following the intervention, representing a 4.14-fold rise (*p* < 0.001). ITS showed that the monthly trend of confirmed rabies cases significantly increased in Bulacan following LFD implementation (coefficient = 0.565, p < 0.001) (Fig. 5). No significant change was observed in the non-implementation provinces of Pampanga (coefficient = 0.020, *p* = 0.823) (Fig. 5A) and Tarlac (coefficient = 0.017, *p* = 0.669) (Fig. 5B), while Zambales, also a non-implementation province, showed a significant decline (coefficient = −0.099, *p* = 0.015) (Fig. 5C). The differences in trends between Bulacan and each comparison province were statistically significant (*p* < 0.01).

### 3.3 Spatial distribution of rabies cases

**Fig. 6:**
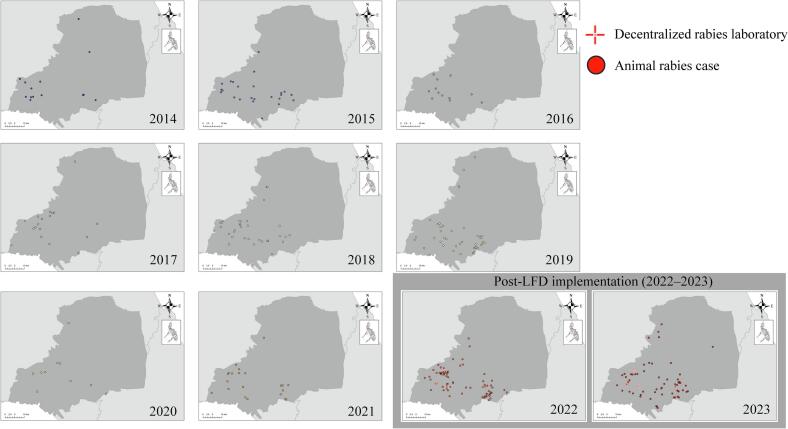
Spatial distribution of confirmed animal rabies cases in municipalities surrounding the decentralized rabies laboratory in Bulacan province, pre- and post-LFD implementation (2014–2023). This figure shows the spatial distribution of DFAT-confirmed animal rabies cases in municipalities surrounding the decentralized rabies laboratory in Bulacan Province during the pre-implementation (2014–2021) and post-implementation (2022–2023) periods. The introduction phase of LFD testing began in October 2021, with full implementation starting in January 2022. Case locations were mapped using individual GPS coordinates recorded in the Rabies Data System, based on data submitted by veterinary officers from municipal, city, and provincial government offices.

Fig. 6 shows the spatial distribution of confirmed animal rabies cases in municipalities surrounding the decentralized rabies laboratory in Bulacan Province, pre- and post-LFD implementation. Following the introduction of LFD testing, the number and geographic spread of confirmed rabies cases increased notably in the areas near the decentralized rabies laboratory.

### 3.4 Response measures following rabies case detection by LFD

Among the 147 LFD-positive cases identified during the observation period, 126 (85.7 %) were recorded in RaDSS, of which 65.9 % were entered within 3 days of diagnosis (Table 1). Among the 126 cases, case investigations were conducted in 69 (54.8 %) cases, while One Health approach, defined as joint case investigations involving collaboration between the local veterinary office, the health office, and the barangay, were performed in only 5.6 %. Response measures such as mass dog vaccination, community awareness activities, and restriction of free-roaming dogs were implemented in 36.5 %, 35.7 %, and 27.8 % of cases, respectively. During the observation period, a total of 129 human bite victims and 99 exposed animals were reported as being exposed to LFD-confirmed rabid animals. In 42.9 % of cases, no human victims were reported, while 48.9 % involved 1–3 bite victims (Table 1). Among the 69 case investigations conducted, 22 unvaccinated human bite victims who had not sought PEP were identified and referred for medical care by the investigation team. At least one unvaccinated human victim was found in 18.8 % of the investigations, with a mean of 0.32 per investigation. Of the 99 exposed animals, 66 (68.8 %) were identified through case investigation. Exposed animals were detected in 54.5 % of the investigations, with an average of 0.96 animals identified per investigation.

**Table 1:** Rabies outbreak responses and follow-up findings after LFD-positive diagnoses at decentralized rabies laboratories.

*N* = 126⁎	Category	n	%
Days from LFD-positive result to RaDSS reporting	Within 3 days	83	65.9 %
	4–14 days	26	20.6 %
	≥15 days	17	13.5 %
Case investigation conducted	Yes	69	54.8 %
	No	57	45.2 %
Mass dog vaccination conducted	Yes	46	36.5 %
	No	80	63.5 %
Information, education and communication campaign conducted	Yes	45	35.7 %
	No	81	64.3 %
One Health approach investigation conducted†	Yes	7	5.6 %
	No	119	94.4 %
Free-roaming dog restriction conducted	Yes	35	27.8 %
	No	91	72.2 %
Number of humans exposed to LFD-confirmed rabid animals	0	54	42.9 %
	1–3	61	48.9 %
	4–6	9	7.1 %
	Unknown	2	1.6 %
Number of animals exposed to LFD-confirmed rabid animals	0	82	65.1 %
	1–4	33	26.2 %
	5–9	2	1.6 %
	≥10	1	0.8 %
	Unknown	8	6.3 %

Abbreviation: RaDSS, Rabies Data Share System.

⁎ Includes only LFD-positive cases reported in RaDSS.

† One Health approach refers to case investigations jointly conducted by the local health office and the veterinary office.

## 4 Discussion

We introduced LFDs into areas lacking local diagnostic capacity and assessed their impact on surveillance performance and field response. This study demonstrated that LFDs showed high diagnostic accuracy in decentralized settings and significantly improved animal rabies surveillance. Furthermore, LFD-triggered case investigations led to the identification of unvaccinated bite victims and exposed animals, demonstrating their practical value in supporting timely public health and veterinary actions.

### 4.1 Diagnostic accuracy of LFDs in decentralized rabies laboratories

Onsite LFDs demonstrated high diagnostic performance, with a sensitivity of 97.0 % and a specificity of 98.2 %. These results align with findings from Zanzibar, which reported 95 % sensitivity using the same ADTEC LFD [20]. A nationwide study using ADTEC LFD in the Philippines also reported 96.3 % sensitivity and 99.7 % specificity [19]. Similar diagnostic performance has been reported in evaluations using the Bionote kit (BioNote Inc., Hwaseong-si, Republic of Korea), with sensitivity and specificity of 96 % and 99 % using samples from India, 95.3 % and 93.3 % in a field study conducted in Chad, and 90 % and 99 % using samples originating from Chad and other countries [16,17,25]. While some LFDs showed low sensitivity, ADTEC and Bionote LFD have demonstrated high accuracy, highlighting the importance of using validated diagnostic kits [19,23,24]. It is also worth noting that better diagnostic performance has been reported in studies conducted in front-line settings using fresh or well-preserved brain samples compared to studies that used long-stored specimens [16,18,[23], [24], [25],^28^,[30], [31], [32]]. Although LFDs are rapid and simple methods, suboptimal specimen processing, particularly inadequate homogenization, remains a potential cause of false negatives [19]. In addition, recent evaluations using both the ADTEC and Bionote kits omitted the dilution step which contributed to improved sensitivity [17,23]. A national evaluation in the Philippines highlighted the need for adequate technical training, emphasizing the importance of specimen handling and biosafety when introducing LFDs in settings without prior rabies diagnostic experience [19].

### 4.2 Surveillance impacts

LFD introduction in Bulacan led to a significant increase in confirmed rabies cases, suggesting improved case detection [33,34]. To adjust for this background trend, we performed longitudinal and ITS analyses comparing Bulacan with three nearby provinces (Pampanga, Tarlac, and Zambales), which showed a significantly greater increase in confirmed cases in Bulacan after LFD implementation than in the control provinces. While previous studies have observed increased case detection following the introduction of LFDs, this is the first study to quantitatively assess the impact of LFD deployment in comparison with non-intervention areas, while adjusting for underlying regional rabies trends [16,20]. In Goa, 209 brain samples were tested with LFDs at the field level, showing 96 % sensitivity, 99 % specificity, and a 56 % positivity rate compared to FAT. Similarly, in Bhutan, field testing of 179 brain samples resulted in 92 % sensitivity, 100 % specificity, and a 64 % positivity rate. However, neither study statistically demonstrated whether the use of LFDs effectively enhanced surveillance [16,18,35]. LFDs were introduced into eight decentralized labs in Namibia, but poor reporting left actual field test numbers unclear, highlighting the need for proper reporting systems when implementing LFDs [32]. The study indicated that in countries like Namibia, where samples could reach a central laboratory within 24 h and results returned within 48–72 h, decentralized LFD testing was likely unnecessary. However, the study did not evaluate field-level experiences or impacts, and thus the effect of introducing LFDs should have been assessed more thoroughly. Rabies case detection in our study increased approximately fourfold following LFD implementation. Similar findings were reported in Zanzibar, where deploying ADTEC LFDs under a standardized framework. This framework, incorporating the RAIDER (Rapid In-field Diagnosis and Epidemiology of Rabies) toolkit to ensure standardized field-level diagnostics and consistent epidemiological reporting, also leveraged real-time data to enable more targeted dog vaccination campaigns and facilitated more timely outbreak responses. These observations underscore the operational value of decentralized diagnostics in strengthening surveillance systems and supporting rabies control in endemic regions [20]. Our study used similar methodologies and intervention approaches but was conducted prior to the Zanzibar study, demonstrating comparable outcomes. Moreover, by incorporating comparisons with surrounding provinces, our analysis provided a more comprehensive evaluation of the impact of decentralized LFD-based surveillance. DRIT is another option for decentralized rabies diagnosis, as demonstrated in Tanzania, Nigeria, and Benin [[36], [37], [38]]. While generally more sensitive than LFDs, DRIT requires electricity and microscopy, making LFDs easier to deploy in field settings.

### 4.3 Impacts and operational challenges of LFD-triggered rabies response activities

In this study, case investigations following LFD-positive results enabled response teams to identify human bite victims who had not received PEP and previously unrecognized exposed animals, underscoring their importance in guiding timely post-exposure interventions to prevent rabies transmission and save lives. The ability to diagnose on-site using LFDs appears to have influenced field-level response and reporting behavior [20]. Previously, obtaining diagnostic results often took days to weeks, but the introduction of LFDs enabled local veterinarians to perform rabies testing onsite and obtain immediate results. There were reports in the study that the ability to obtain immediate results motivated local veterinarians to take a more active role in rabies control. Increased reporting of suspected animals by community members and local authorities was also documented in areas following LFD-positive detections. These operational benefits suggest that real-time diagnostic feedback at the local level can improve engagement and prompt action in rabies surveillance systems.

Integrated Bite Case Management (IBCM) has been shown to enhance rabies surveillance [[39], [40], [41]], but in Southeast Asia, where animal bite incidence is high, field implementation for all high risk bite cases is often impractical. While focusing on high-risk bites could optimize efforts, this is challenging given that many human rabies cases, such as in the Philippines, result from minor pet bites [33]. Incorporating LFD testing into the IBCM framework offers a more effective strategy for rabies control. Efficient identification of suspected animals or those found dead of unknown cause, combined with on-site screening, enables the rapid detection of more rabid animals and strengthens surveillance. The rapid diagnosis by LFD makes it possible for local teams to implement prompt and targeted control measures based on confirmed cases.

However, challenges remain in implementing a One Health approach for rabies outbreak response and case management, involving local health and veterinary sectors jointly conducting rapid case investigations. Under the current institutional framework in the Philippines, the animal health sector is responsible for animal rabies cases, while the public health sector responds to human cases. The lack of joint implementation between sectors often leads to parallel and uncoordinated investigations, with limited exchange of critical information in a timely manner. In this study, among the 69 case investigations conducted, only 7 (10.1 %) involved collaboration between health and veterinary authorities, illustrating the absence of an established mechanism for joint response. As a result, opportunities for timely and integrated interventions are frequently missed. To enable effective One Health outbreak responses, a legal framework and institutional structure must be developed to formalize multisectoral coordination and clearly define the roles and responsibilities of each sector.

### 4.4 Limitation

This study has several limitations. First, although we documented the implementation of case investigations and some immediate actions, we were not able to evaluate the effectiveness of these responses in preventing onward transmission. Data on follow-up outcomes of exposed individuals and animals were not available, limiting our ability to assess the true public health and veterinary impact of the interventions. Second, the selection of the LFD implementation area was not randomized. Local administrative decisions influenced site selection, and pre-existing differences in surveillance capacity or political will may have introduced selection bias. Apart from the introduction of LFDs, the presence of proactive local veterinarians likely played a critical role in case detection, which may have influenced the observed outcomes. Third, the study was conducted in a single province in the Philippines. The findings may not be directly generalizable to other regions with different health systems, infrastructure, or levels of rabies endemicity. Fourth, a precise cost analysis was not feasible, as the implementation was supported not only by the research budget but also by external contributions from both the DOH and the DA. Finally, we were unable to track the long-term outcomes of human and animal contacts identified through case investigations. As such, the study could not directly demonstrate reductions in rabies transmission attributable to the interventions.

Through our LFD implementation, our experience indicated that to effectively enhance rabies surveillance through the use of LFDs and support sustained rabies control efforts, the following components are critical: (1) adequate training of veterinary staff in specimen processing, PPE, and biosafety; (2) establishing reporting processes and training following onsite testing; (3) strategic selection of implementation sites based on geographical access and human rabies incidence; (4) sensitization of the public and health sector (including animal bite treatment centers) to encourage reporting of animal bites and deaths to veterinary services; and (5) rapid and effective outbreak responses under a One Health approach following case detection.

## 5 Conclusion

LFD-based rabies diagnosis in decentralized laboratory settings demonstrated high diagnostic accuracy, and its introduction significantly strengthened surveillance by leading to a nearly fourfold increase in confirmed rabies case detection. Case response following LFD confirmation also enabled the identification of unvaccinated bite victims and exposed animals. Strengthening One Health coordination for response activities and ensuring the sustainability of LFD-based surveillance should be priorities for future research.

## CRediT authorship contribution statement

**Nobuo Saito:** Writing – review & editing, Writing – original draft, Visualization, Validation, Supervision, Software, Resources, Project administration, Methodology, Investigation, Formal analysis, Data curation, Conceptualization. **Patricia T. Lacanilao:** Writing – review & editing, Project administration, Investigation. **Alyssa M. Garcia:** Writing – review & editing, Project administration, Investigation. **Karren L. Inton:** Writing – review & editing, Project administration, Investigation. **Jaira D. Mauhay:** Writing – review & editing, Project administration, Investigation. **Voltaire G. Basinang:** Writing – review & editing, Resources, Project administration, Investigation. **Lea G. Fernando:** Writing – review & editing, Resources, Project administration, Investigation. **Benedict T. Bernardo:** Writing – review & editing, Resources, Project administration, Investigation. **Roel G. Dela Cruz:** Writing – review & editing, Resources, Project administration, Investigation. **Arvin H. Agapito:** Writing – review & editing, Resources, Project administration, Investigation. **Arby B. Banaag:** Writing – review & editing, Resources, Project administration, Investigation. **Gladys M. Bernardo:** Writing – review & editing, Resources, Project administration, Investigation. **Jonarel M. Andres:** Writing – review & editing, Resources, Project administration, Investigation. **Geraldine M. San Juan:** Writing – review & editing, Resources, Project administration, Investigation. **Edwin P. Tecson:** Writing – review & editing, Resources, Project administration, Investigation. **Annie M. Balingit:** Writing – review & editing, Resources, Project administration, Investigation. **Joely T. Ongtangco:** Writing – review & editing, Supervision, Methodology, Conceptualization. **Maria G. Lagayana:** Supervision, Methodology, Conceptualization. **Jeffrey L. Cruz:** Writing – review & editing, Supervision, Methodology, Conceptualization. **Shella G. Oridinario:** Writing – review & editing, Supervision, Methodology, Conceptualization. **Catalino S. Demetria:** Writing – review & editing, Supervision, Methodology, Conceptualization. **Daria L. Manalo:** Writing – review & editing, Supervision, Methodology, Conceptualization. **Beatriz P. Quiambao:** Writing – review & editing, Supervision, Methodology, Conceptualization. **Kazunori Kimitsuki:** Writing – review & editing, Supervision, Methodology, Conceptualization. **Akira Nishizono:** Writing – review & editing, Validation, Supervision, Methodology, Funding acquisition, Conceptualization.

## Consent for publication

Not applicable.

## Author statement

All authors have read and approved the final version of the manuscript. All authors meet the authorship criteria of the International Committee of Medical Journal Editors (ICMJE) and agree with the authorship order. The authors declare no additional statements.

## Ethic approval and consent to participate

Not applicable.

## Funding

This work was supported by a JICA/AMED SATREPS (Science and Technology Research Partnership for Sustainable Development) (https://www.jst.go.jp/global/english/index.html) for “The establishment of the One Health prevention and treatment network model for the elimination of rabies in the Philippines” (No.17823721) to AN. This work was also partially supported by the Japan Society for the Promotion of Science (JSPS) Kakenhi Grant-in-Aid for Scientific Research (Grant number 24KK0174) to Nobuo Saito. The funders played no role in study design; in the collection, analysis, and interpretation of data; in the writing of the report; and in the decision to submit the paper for publication.

## Declaration of competing interest

The authors declare no competing interests.

The authors declare that they have no known competing financial interests or personal relationships that could have appeared to influence the work reported in this paper.

## Data Availability

The data that support the findings of this study are available from the corresponding author upon reasonable request.

## Appendix A Supplementary data

Table S1. STARD (Standards for Reporting Diagnostic accuracy studies) checklist. A completed STARD checklist summarizing adherence to reporting standards for diagnostic accuracy studies in this manuscript. Table S2. Discrepant results between onsite LFD, regional laboratory DFAT, and LN34 RT-qPCR. A summary of all samples with discordant results among onsite LFD testing, regional DFAT confirmation, and molecular testing using LN34 RT-qPCR. Image 1
