# Supplementary material for: LFD implementation uncovers hidden rabies burden: Pre- and post-implementation analysis and comparison with non-implementation sites in the Philippines
Source: One Health. 2025 Nov 19;21:101281. doi: 10.1016/j.onehlt.2025.101281 (PMC12681732; doi:10.1016/j.onehlt.2025.101281)
Supplement: Supplementary file 1 — Table S1. STARD (Standards for Reporting Diagnostic accuracy studies) checklist. A completed STARD checklist summarizing adherence to reporting standards for diagnostic accuracy studies in this manuscript. Table S2. Discrepant results between onsite LFD, regional laboratory DFAT, and LN34 RT-qPCR. A summary of all samples with discordant results among onsite LFD testing, regional DFAT confirmation, and molecular testing using LN34 RT-qPCR. [file mmc1.pdf]

Supplementary tables

Table S1 Table. STARD (Standards for Reporting Diagnostic accuracy studies) checklist

Section & Topic	No	Item	Reported on page #
**TITLE OR ABSTRACT**			
	**1**	Identification as a study of diagnostic accuracy using at least one measure of accuracy (such as sensitivity, specificity, predictive values, or AUC)	Page 4-5, Abstract
**ABSTRACT**			
	**2**	Structured summary of study design, methods, results, and conclusions (for specific guidance, see STARD for Abstracts)	Page 4-5 abstract
**INTRODUCTION**			
	**3**	Scientific and clinical background, including the intended use and clinical role of the index test	Page 6-7
	**4**	Study objectives and hypotheses	Page 7-8
**METHODS**			
Study design	**5**	Whether data collection was planned before the index test and reference standard were performed (prospective study) or after (retrospective study)	Page 8-9
Participants	**6**	Eligibility criteria	Page 10-11
	**7**	On what basis potentially eligible participants were identified (such as symptoms, results from previous tests, inclusion in registry)	Page 10-11, Page 8-9
	**8**	Where and when potentially eligible participants were identified (setting, location and dates)	Page 8-9
	**9**	Whether participants formed a consecutive, random or convenience series	Page 10-11
Test methods	**10a**	Index test, in sufficient detail to allow replication	Page 10-11
	**10b**	Reference standard, in sufficient detail to allow replication	Page 11-12
	**11**	Rationale for choosing the reference standard (if alternatives exist)	Page 6-7
	**12a**	Definition of and rationale for test positivity cut-offs or result categories of the index test, distinguishing pre-specified from exploratory	Page 10-11
	**12b**	Definition of and rationale for test positivity cut-offs or result categories of the reference standard, distinguishing pre-specified from exploratory	Page 11-12
	**13a**	Whether clinical information and reference standard results were available to the performers/readers of the index test	Page 10-11

	**13b**	Whether clinical information and index test results were available to the assessors of the reference standard	Page 10-11
Analysis	**14**	Methods for estimating or comparing measures of diagnostic accuracy	Page 12, 15-16
	**15**	How indeterminate index test or reference standard results were handled	Page 15-16
	**16**	How missing data on the index test and reference standard were handled	Page 15-16
	**17**	Any analyses of variability in diagnostic accuracy, distinguishing pre-specified from exploratory	Page 16-17
	**18**	Intended sample size and how it was determined	Not specified.
**RESULTS**			
Participants	**19**	Flow of participants, using a diagram	Figure 2
	**20**	Baseline demographic and clinical characteristics of participants	Page 15-16
	**21a**	Distribution of severity of disease in those with the target condition	Not specified.
	**21b**	Distribution of alternative diagnoses in those without the target condition	Not specified.
	**22**	Time interval and any clinical interventions between index test and reference standard	Page 10-11
Test results	**23**	Cross tabulation of the index test results (or their distribution) by the results of the reference standard	Page 15-16, 41-42 Figure 2
	**24**	Estimates of diagnostic accuracy and their precision (such as 95% confidence intervals)	Page 15-16,Figure 2
	**25**	Any adverse events from performing the index test or the reference standard	Not applicable.
**DISCUSSION**			
	**26**	Study limitations, including sources of potential bias, statistical uncertainty, and generalisability	Page 27-28
	**27**	Implications for practice, including the intended use and clinical role of the index test	Page 28-29
**OTHER INFORMATION**			
	**28**	Registration number and name of registry	Not applicable.
	**29**	Where the full study protocol can be accessed	Not specified.
	**30**	Sources of funding and other support; role of funders	Page 30-31

Table S2. Discrepant Results Between Onsite LFD, Regional Laboratory DFAT, and LN34 RT-qPCR

ID	Decentralized rabies laboratories	Species	Onsite LFD results	Regional Lab DFAT results	Regional lab LFD results	LN34 RT-qPCR results	CT value Brain sample LN34 RT-qPCR	Interpretation
50	Pulilan	Dog	Negative	Positive	Negative	Positive	29	Onsite LFD false-negative
83	Pulilan	Dog	Negative	Positive	Negative	Negative	Undetermined	Possible false-positive DFAT result
99	Guiguinto	Cat	Positive	Negative	Negative	Negative	Undetermined	Onsite LFD false-positive. Possible misinterpretation during field testing.
197	San Jose Del Monte	Dog	Negative	Positive	Not Performed	Negative	Undetermined	Possible false-positive DFAT result
205	San Jose Del Monte	Dog	Negative	Positive	Positive	Positive	14	Onsite LFD false-negative

LFD: lateral flow device; DFAT: direct fluorescent antibody test; RT-qPCR: real-time reverse transcription PCR; CT: cycle threshold.
